# The Application of Metabolomics in Hyperlipidemia: Insights into Biomarker Discovery and Treatment Efficacy Assessment

**DOI:** 10.3390/metabo14080438

**Published:** 2024-08-06

**Authors:** Mohammad Alwahsh, Rahaf Alejel, Aya Hasan, Haneen Abuzaid, Tariq Al-Qirim

**Affiliations:** Faculty of Pharmacy, Al-Zaytoonah University of Jordan, Amman 17138, Jordan; alejelrahaf@gmail.com (R.A.); ayaahasan97@gmail.com (A.H.); haneen.abuzaid@zuj.edu.jo (H.A.); tariq.qirim@zuj.edu.jo (T.A.-Q.)

**Keywords:** biomarkers, hyperlipidemia, metabolic pathway, metabolites, metabolomics

## Abstract

Hyperlipidemia is a lipid metabolism disorder that refers to increased levels of total triglycerides (TGs), cholesterol (TC), and low-density lipoprotein-cholesterol (LDL-C) and decreased levels of high-density lipoprotein-cholesterol (HDL-C). It is a major public health issue with increased prevalence and incidence worldwide. The ability to identify individuals at risk of this disorder before symptoms manifest will facilitate timely intervention and management to avert potential complications. This can be achieved by employing metabolomics as an early detection method for the diagnostic biomarkers of hyperlipidemia. Metabolomics is an analytical approach used to detect and quantify metabolites. This provides the ability to explain the metabolic processes involved in the development and progression of certain diseases. In recent years, interest in the use of metabolomics to identify disease biomarkers has increased, and several biomarkers have been discovered, such as docosahexaenoic acid, glycocholic acid, citric acid, betaine, and carnitine. This review discusses the primary metabolic alterations in the context of hyperlipidemia. Furthermore, we provide an overview of recent studies on the application of metabolomics to the assessment of the efficacy of traditional herbal products and common lipid-lowering medications.

## 1. Introduction

Hyperlipidemia is a complex disease defined as an abnormality in lipid metabolism marked by elevated levels of total cholesterol (TC), triglycerides (TG), and low-density lipoprotein-cholesterol (LDL-C) and/or decreased levels of high-density lipoprotein-cholesterol (HDL-C) in the peripheral blood. It is considered a major risk factor for cardiovascular diseases (CVDs) [1]. Hyperlipidemia can be broadly categorized into two main types based on its underlying etiology: primary and secondary hyperlipidemia [2]. Primary hyperlipidemia, also known as familial hyperlipidemia, is a form of hyperlipidemia caused by genetic abnormalities [3]. Genetic abnormalities can be monogenic or polygenic in nature. Familial hyperlipidemia is classified into five types according to the Fredrickson classification: I, II, III, IV, and V, based on the elevated lipoprotein class [4,5]. In contrast, secondary hyperlipidemia, also known as acquired hyperlipidemia, is a type of hyperlipidemia caused by an underlying factor that leads to abnormalities in lipid metabolism and elevated plasma lipid levels [6]. Hyperlipidemia risk factors can be categorized as modifiable or non-modifiable [7]. Modifiable risk factors include the following: a diet that involves a high consumption of saturated and trans fats; a low intake of fruits, vegetables, and fiber; certain drugs; alcohol consumption; smoking; metabolic disorders, such as diabetes (both types 1 and 2) and polycystic ovary syndrome; obesity and weight gain; physical inactivity; high blood pressure; and poor sleep quality [3]. Non-modifiable risk factors include aging; sex, where men are generally at a higher risk; genetics, such as a family history of hyperlipidemia; endocrine disorders, including hypothyroidism and Cushing’s syndrome; and renal failure [3,8]. CVDs are a group of diseases that affect the heart and circulatory system. They include coronary heart diseases, cerebrovascular diseases, and myocardial infarction [9,10]. According to the World Health Organization (WHO), CVDs are the major causes of mortality and morbidity globally. They are responsible for approximately one-third of all deaths worldwide by 2021, representing approximately 20.5 million people [11], which is expected to increase to 22 million by 2030 [12]. The Global Burden of Disease study reported that approximately 9.2 million deaths in 2022 were attributed to ischemic heart disease [13]. Although the development of CVDs can be attributed to several factors, atherosclerosis is the primary cause of CVDs globally [14]. Atherosclerosis is a complex condition involving a multitude of factors including lipid accumulation, inflammation, endothelial dysfunction, vascular smooth muscle cell proliferation, and calcification [15]. These processes ultimately lead to the development of atherosclerotic plaques, which, in turn, lead to a narrowing of the arteries, decreased blood flow, and an increased risk of cardiovascular complications [15,16]. Hyperlipidemia is a major health concern that plays an important role in the development of CVDs and is considered a major risk factor for the development of atherosclerosis [17,18]. Hyperlipidemia often presents with no obvious symptoms, making early detection difficult and increasing the risk of serious complications. Hence, there is an urgent need for the early detection and management of hyperlipidemia to reduce the risk of the development of CVDs [19]. Metabolomics provides a more comprehensive analysis of metabolic alterations and offers a tool for the discovery of novel diagnostic biomarkers [20]. This comprehensive approach sheds light on the disease mechanisms, allowing for an earlier and more accurate diagnosis compared to traditional lipid profiles, which often focus on a subset of lipid characteristics. Furthermore, metabolomics can detect dynamic changes in the metabolism, enabling personalized treatment methods that are not possible using traditional lipid parameters [21]. The initial management of hyperlipidemia involves the alteration of modifiable risk factors, including a healthy diet, physical activity, smoking cessation, and weight loss [22]. Lipid-lowering medications are initiated in conjunction with lifestyle modifications [23,24]. Commonly prescribed lipid-lowering medications include 3-hydroxy-3-methylglutaryl coenzyme A (HMG-CoA) reductase inhibitors (statins), peroxisome proliferator-activated receptor α agonists (fibrates), bile acid sequestrants, nicotinic acid, and cholesterol absorption inhibitors (Ezetimibe) [25]. 

Metabolomic studies of hyperlipidemic models have identified the potential biomarkers that expand our understanding of the mechanisms underlying the pathogenesis and progression of hyperlipidemia and might enable the diagnosis of hyperlipidemia at an earlier stage. This review aims to provide an overview of the findings of current animal and human metabolomics-based studies in the past 10 years in association with hyperlipidemia, highlight their contributions to the field, and identify possible areas for future applications. Additionally, despite several recent applications of metabolomics in evaluating the anti-hyperlipidemic activity of traditional herbal agents and common lipid-lowering medications, there is still a shortage of reviews that address these findings. Hence, in this review, we will address the importance of these therapeutic agents and provide a better understanding of their underlying mechanisms of action. 

## 2. Metabolomics

Metabolomics is the comprehensive analysis of the small molecules called metabolites produced by biological samples, including cells, tissues, organs, and biofluids, by applying analytical methods such as mass spectrometry (MS) and Nuclear Magnetic Resonance (NMR) spectroscopy [26,27]. Metabolites are the intermediate byproducts of metabolic processes that represent the final downstream products. Thus, any underlying changes in genomics, transcriptomics, or proteomics can be reflected in metabolic alterations [28]. The field of metabolomics has evolved rapidly and holds promise for use as a powerful tool to help understand the interactions within a biological system and enable the search for metabolic biomarkers that can function as indicators of disease progression or therapeutic intervention efficacy [26,29]. Metabolomic studies can be divided into two approaches: untargeted and targeted approaches. Untargeted metabolomics is the comprehensive profiling of all the detectable metabolites in a sample and is usually intended for the discovery of new biomarkers. The targeted metabolomics approach mainly focuses on the quantification of metabolites of interest to validate and further investigate a specific group of metabolites [30,31]. Over the last few years, metabolic profiling has been employed in several studies to improve our understanding of the pathogenesis of hyperlipidemia. In addition, it has been applied in various studies to evaluate the potential anti-hyperlipidemic activity of many agents [32,33]. Metabolomics-based studies focus on the identification of differential metabolites, as well as the investigation of related metabolic pathways, as any abnormalities in metabolite levels would reflect these pathways [34]. After identifying a set of significant metabolites that could serve as potential biomarkers, the next step is to investigate the relationships between these metabolites and their metabolic pathways. This includes the study of the synthesis and degradation processes related to alterations in metabolites. By understanding these connections, we can elucidate the possible mechanisms that lead to alterations in metabolite levels. This knowledge can also reveal how metabolic dysfunction contributes to disease development and progression [35,36]. 

### 2.1. Metabolomics Techniques 

Several analytical methods have been employed in the field of metabolic profiling to identify and quantify a set of metabolites [37]. The most frequently used analytical techniques are NMR and MS [38], including ultraperformance liquid chromatography–tandem mass spectrometry (UPLC-MS/MS) [39], liquid chromatography–mass spectrometry (LC-MS) [40,41], liquid chromatography–tandem mass spectrometry (LC-MS/MS), and gas chromatography–mass spectrometry (GC-MS) [42]. The reproducibility and non-destructive properties of NMR analysis and the high sensitivity and selectivity of MS analysis have made these two techniques the most employed in sample analysis in metabolomics [43,44,45]. MS is typically coupled with separation techniques such as gas chromatography (GC) and liquid chromatography (LC). This coupling of MS with GC and LC allows the separation of metabolites based on their different chromatographic properties and increases their selectivity, contributing to their superiority in targeted metabolomics [46]. Other techniques, including Fourier-transform infrared (FTIR) spectroscopy [39], Raman spectroscopy [47], and molecularly imprinted polymer (MIP)-based electrosensors [48], have also been used for the sample analysis. Each analytical technique has its own advantages and disadvantages, as listed in Table 1. Hence, the choice of the analytical technique depends on many factors, including the type and amount of the sample; the concentration and properties of the metabolites detected; the experimental design; the aim of the metabolomic study; and, more specifically, the question being explored in the experiment [44,49]. Moreover, although each analytical technique has several strengths, no single method is considered ideal for the detection of all the metabolites in a sample. A multiplatform approach that combines analytical techniques provides a more comprehensive and complementary analysis of the metabolome [50,51]. In the context of hyperlipidemia metabolomics-based studies, both NMR-based metabolomic research and MS-based metabolomic research have been conducted to investigate hyperlipidemia and are discussed in this review. Employing advanced analytical techniques enables the detection of a wide range of metabolites, which, in turn, provides comprehensive insights into the pathophysiological processes underlying hyperlipidemia and sheds light on possible therapeutic targets. 

**Figure 1:**
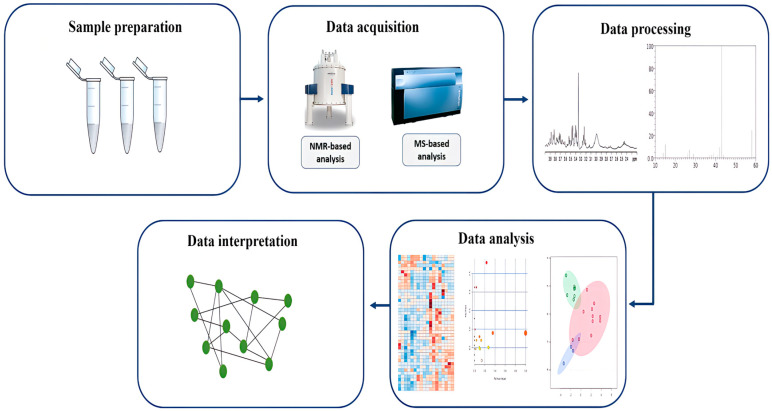
Metabolomics workflow.

The metabolomic data undergo several steps, as shown in Figure 1. After data acquisition through an analytical method of choice, an extensive amount of complex raw data is obtained, which is then preprocessed using certain software tools. The data are then processed and normalized to reduce bias and analyzed using univariate and multivariate statistical analysis methods to identify the statistically significant metabolites that differentiate between the studied groups. All these steps allow the efficient interpretation of the data and lead to potentially valuable results [52,53]. 

**Table 1:** Advantages and limitations of the most commonly used analytical techniques in metabolomics.

Analytical Technique	Advantages	Limitations	References
Nuclear Magnetic Resonance (NMR) spectroscopy	- Simple sample preparation - Short analysis time - Robustness - High reproducibility - Non-destructive - Can be applied on a variety of samples, including intact tissues - High throughput method	- Lower sensitivity compared to MS - Requires larger sample volume - NMR spectrometers are more expensive compared to mass spectrometers - Challenges in data interpretation and integration across platforms	[46]
Liquid chromatography–mass spectrometry (LC-MS)	- High selectivity - High sensitivity (picomolar and nanomolar levels) - Needs small amounts of sample volume (a few microliters) - Higher resolution compared to NMR - Sample derivatization is not needed - Suitable for analysis of wide range of metabolites, such as thermolabile metabolites	- Destructive technique - Requires tissue extraction from tissue samples - Additional chromatography time - Limited libraires available	[44,54]
Gas chromatography–mass spectrometry (GC-MS)	- High selectivity - High sensitivity - Needs small amounts of sample volume (a few microliters) - Higher resolution compared to NMR Large spectral libraries	- Destructive technique - Requires tissue extraction from tissue samples - Additional chromatography time - Not suitable for non-volatile and thermolabile metabolites - Requires chemical derivatization	[44,54]
Fourier-transform infrared (FTIR) spectroscopy (less common)	- Rapid analysis - Molecular fingerprints - Good predictive model - High specificity	- Does not identify metabolites	[39]

### 2.2. The Need for Biomarkers in Hyperlipidemia

The primary focus of metabolic profiling is the identification of the biomarkers that are the biological indicators of normal physiological, pathological, or therapeutic responses [36,55]. These biomarkers have notable benefits not only in disease diagnosis but also in disease progression, classification, and treatment response monitoring [56]. Biomarkers can be categorized into several types based on their intended applications, such as predictive, prognostic, monitoring, and diagnostic [55]. The traditional clinical markers of hyperlipidemia (TC, TG, LDL-C, and HDL-C) are routinely used as screening tools to evaluate cardiovascular risk and to monitor the response to lipid-lowering therapy. However, these markers have been proposed to have low sensitivity and specificity [29,57] and fail to fully capture the risk of CVDs [58,59]. This amplifies the necessity for the discovery of the sensitive and specific biomarkers that could enable the early detection of hyperlipidemia. Metabolomics-based biomarkers have the potential to detect diseases at an early stage, even in the asymptomatic phase, because metabolite changes and disruptions can develop at a very early stage of the disease development [44].

## 3. Metabolomics for the Identification of the Potential Biomarkers Associated with Hyperlipidemia

**Figure 2:**
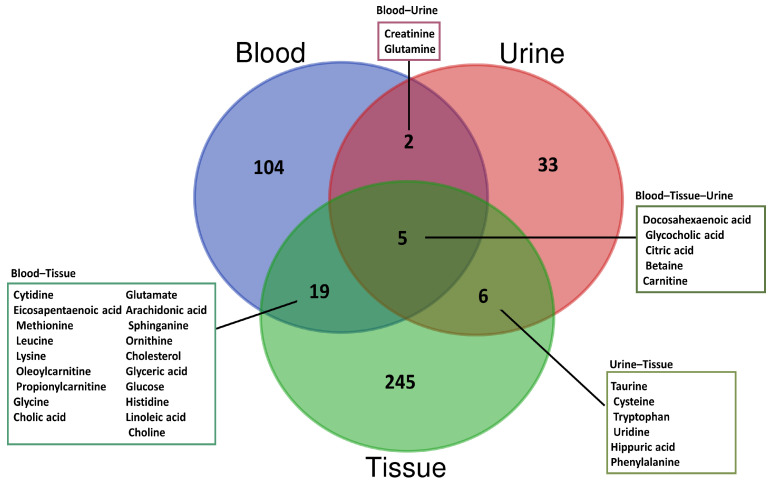
Venn diagram of altered metabolites shared among different sample types of hyperlipidemia models. Pink: urine samples; blue: blood samples; and green: tissue samples.

Understanding the changes within the body’s metabolic system is crucial, because hyperlipidemia is a systemic disease that influences the metabolism of various endogenous metabolites [60]. While the current clinical markers of hyperlipidemia may provide a general idea of the disease state, the growing need for novel biomarkers that can detect abnormalities in the body’s metabolic processes and address the underlying pathophysiological mechanisms has inspired the use of metabolomics in research [21]. In the last ten years (2014–2024), several metabolomics studies have been conducted on a variety of biological samples, encompassing both tissues and various biofluids. The detailed search strategy for the articles included in this review is summarized in Table 2. The primary objectives of these studies were twofold: to discover the metabolic biomarkers indicative of hyperlipidemia and to elucidate the intricate metabolic alterations that occur during the development of this condition. In this review, we provide a concise overview of the findings of published articles, focusing primarily on the discovery of biomarkers in different sample types. Among these studies, three were human metabolomics studies and the remainder were animal metabolomics studies. A synopsis of metabolomic studies for the discovery of the candidate biomarkers associated with hyperlipidemia is presented in Table 3. The common differential metabolites among different sample types identified by human and animal metabolomics studies are represented using a Venn diagram (Figure 2).

### 3.1. Blood-Based Metabolomics

The discovery of blood-based biomarkers is of great interest because they offer a convenient and minimally invasive tool for the early diagnosis of the disease [61,62]. Metabolomics studies in hyperlipidemic models have consistently shown elevated levels of TC, TG, and LDL-C in animals [33,63] and human subjects [64,65], as well as the depletion of HDL-C levels [63,65]. Over the years, several clinical studies have employed a metabolomic approach to advance the understanding of hyperlipidemia. In 2016, a clinical trial was conducted to analyze serum samples from 71 patients with hyperlipidemia and 100 healthy individuals using an UPLC-MS approach [21]. A total of 37 metabolites, including many lipids and fatty acids, were identified (Table 3). In addition, 11 metabolic pathways were found to be associated with hyperlipidemia, for example, linoleic acid metabolism and glycerophospholipid metabolism, which are considered the most important metabolic pathways [21]. 

An analysis of the plasma samples from 125 children divided into groups with different cholesterol levels was performed using ^1^H-NMR in a prospective cross-sectional controlled study [66]. Elevated levels of tyrosine, glutamic acid, ornithine, lysine, alanine, creatinine, oxoglutaric acid, and creatine were detected in the blood of the high and borderline cholesterol-level groups (Table 3). Of the identified differential metabolites, tyrosine and glutamic acid were found to have the highest importance at different cholesterol levels. The elevation of the non-essential amino acid tyrosine is suggested to stimulate liver metabolism and increase lipid synthesis, whereas a positive correlation between glutamic acid and cholesterol could be linked to increased visceral fat accumulation, which is considered a risk factor for dyslipidemia. The findings of this study emphasize the involvement of carbohydrate and amino acid metabolism in lipid metabolism and hypercholesterolemia. Moreover, high levels of ornithine were detected in this study. Another study on the hypercholesterolemia metabolic profile consistently revealed elevated ornithine levels in the plasma of Wistar male rats (Table 3), as well as numerous disturbances in the metabolic pathways resulting from the high-cholesterol feeding, including impairments in the tricarboxylic acid (TCA) cycle and beta-oxidation; changes in the lipid metabolites affecting glycerophospholipids, sphingolipid species, and free polyunsaturated fatty acids; elevated levels of glucogenic and ketogenic amino acids; and alterations in bile acid and vitamin D levels, all of which shed light on the underlying metabolic changes in hypercholesterolemia [67]. In another study, ultraperformance liquid chromatography–quadrupole time-of-flight mass spectrometry (UPLC-Q-TOF-MS) was used by Wu et al. to analyze the serum and urine samples of a high-fat diet (HFD)-induced hyperlipidemia rat model [68]. A total of nineteen potential biomarkers associated with hyperlipidemia were identified, as represented in Table 3, nine of which were altered in the serum of HFD rats, including elevated levels of succinic acid, cholic acid, C16 Sphinganine, and Sphinganine and lower levels of five unsaturated fatty acids (stearidonic acid, linoleic acid, 8,11-eicosadiynoic acid, eicosapentaenoic acid, and docosahexaenoic acid (DHA)). Lower levels of unsaturated fatty acids have been reported to be indicators of increased peroxidation and oxidative stress. In addition, one observation in this study was the elevated levels of succinic acid in the serum of HFD rats, which can be attributed to increased fat catabolism, which, in turn, disturbs the TCA cycle and leads to abnormal energy metabolism. This is consistent with the findings of a human experiment reporting metabolic changes in TCA cycle intermediates in hyperlipidemic subjects in fasting and postprandial states, reflecting a clear disturbance in energy metabolism in hyperlipidemia [69]. 

A metabonomic approach was used to investigate the metabolic changes that occur during hyperlipidemia development at nine time points. An analysis of the plasma samples from 90 mice divided into high-fat diet and control groups at nine time points was conducted using the NMR analytical method [70]. Eleven differential metabolites were identified as potential biomarkers, as shown in Table 3, including glycerol, glucose, betaine, glutamate, arginine, and leucine, which were significantly altered in the plasma of hyperlipidemic mice from the beginning to the end of the experiment. In addition, a correlation analysis between the altered metabolites and previously identified biomarkers using the GC-MS method [71] revealed a strong correlation between valine and C22:6. These findings reported the associations of alanine, aspartate, and glutamate metabolism and of D-glutamine and D-glutamate metabolism, as well as arginine biosynthesis, in the pathophysiological process of hyperlipidemia. 

Previous studies have demonstrated that amino acid levels are usually altered during the development of hyperlipidemia. A recent study focused on the role of eight amino acids in hyperlipidemia, using an LC-MS-based targeted metabolomic approach [63]. It was found that the serum levels of alanine, arginine, lysine, methionine, serine, tyrosine, and valine were significantly lower in the hyperlipidemic rats than in the healthy group (Table 3). This imbalance in amino acid metabolism has been reported to be related to the consumption of proteins owing to the metabolic perturbations induced by hyperlipidemia. In particular, a disturbance was observed in glucose metabolism, which was reflected in the lower levels of valine, while an abnormal energy metabolism was linked to the depletion of alanine and serine and an altered lipid metabolism, which was illustrated by the lower levels of tyrosine and methionine and oxidative stress, which was suggested to be linked to depleted levels of lysine and arginine. 

### 3.2. Urine-Based Metabolomics 

In addition to being a noninvasive approach, urine samples can be collected in large quantities, making them one of the most preferred biofluids for biomarker discovery [72]. The disturbance of fatty acid, amino acid, and nucleoside metabolism in diet-induced hyperlipidemia has been reported in a urinary metabolomic-based study [73]. In this study, urine samples from eight diet-induced hyperlipidemia rats and eight healthy controls were analyzed using ultraperformance liquid chromatography coupled with quadrupole time-of-flight synapt high-definition mass spectrometry (UPLC Q-TOF/HDMS), and 16 candidate biomarkers were identified. Among the identified biomarkers, the levels of octadecanamide, oleamide, tryptophan, ursodeoxycholic acid, creatinine, ascorbalamic acid, 3-methyluridine, indole-3-carboxylic acid, and tryptophyl-tyrosine were higher, while the levels of citric acid, adenosine 2′,3′-cyclic phosphate, 3-O-methyldopa, proline, 1-methyladenosine, phenylalanine, and 5-methylcytosine were lower than those in the healthy controls, as shown in Table 3. The findings of this study elucidated the pathophysiological mechanisms underlying diet-induced hyperlipidemia. 

As mentioned earlier, a study by Wu et al. identified 19 metabolites as the biomarkers of hyperlipidemia, which are mainly involved in amino acid, energy, lipid, and nucleotide metabolism [68]. Of these biomarkers, 10 were altered in the urine samples of HFD rats. Higher levels of DHA, 3-methyluridine, uridine, L-isoleucine, and phenyl lactic acid were observed in the HFD rats, whereas the levels of hippuric acid, taurine, L-cysteine, norepinephrine, and L-carnitine were lower than those in the healthy controls.

Many metabolomic studies have linked the pathogenesis of hyperlipidemia to inflammation, oxidative stress, and ongoing amino acid metabolism. Elevated circulating lipid levels have been linked to oxidative stress and an imbalance in the oxidant/antioxidant capacity ratio [74]. Abnormally high lipid and fat catabolism could lead to excessive fatty acid levels in the blood circulation, surpassing the albumin-binding capacity. Hence, elevated free fatty acid levels are associated with oxidative stress and mitochondrial dysfunction [74]. These changes were documented in a clinical trial employing UPLC-Q-TOF/MS technology to analyze the urine samples from 60 hyperlipidemic patients compared with 60 normal control participants [64]. This study identified 22 differential metabolites as the potential biomarkers of hyperlipidemia pathogenesis. The selected biomarkers were mainly associated with amino acid metabolism, energy metabolism, oxidative stress, nucleotide metabolism, steroid hormone metabolism, inflammatory reactions, and the metabolism of intestinal flora. Four metabolic pathways were reported to be closely involved in hyperlipidemia development with high impact values: alanine, aspartate, and glutamate metabolism; arginine and proline metabolism; lysine degradation; and phenylalanine metabolism. Of the 22 potential biomarkers shown in Table 3, metabolites, mainly L-glutamine, L-proline, N-acetylglutamic acid, glutaric acid, and phenylacetic acid, were involved in the metabolic pathways with the highest impact value.

### 3.3. Tissue-Based Metabolomics

The use of biofluid samples for metabolic profiling could be considered a more convenient approach for the investigation of a disease and the discovery of biomarkers, yet tissue-based metabolic profiling could offer greater clarity on the altered metabolic processes and detailed insights into the mechanisms of disease pathogenesis [75]. A recent study conducted multi-organ metabolomic analysis using an MS platform (LC-MS/MS and GC-MS) in HFD-fed apolipoprotein E-deficient (ApoE−/−) mice [76]. Significantly altered levels of 70 metabolites in the aorta, 181 metabolites in the heart, 145 metabolites in the liver, and 172 metabolites in the plasma were observed in HFD-fed ApoE−/− mice compared with those in wild-type mice (Table 3). The findings of this study revealed the possibility that metabolome reprogramming in early hyperlipidemia occurs in multiple tissues. In addition, of the upregulated altered metabolites, several metabolites were specifically upregulated in each analyzed tissue. The metabolites observed in the heart and plasma samples were considered the most unique, indicating that the unique metabolites in a certain tissue cannot be transported and shared with other tissues. This confirmed the hypothesis that metabolic reprogramming exhibits tissue specificity. 

By employing mass spectrometry imaging (MSI), alterations in the metabolites in the liver of a high-lipid rat model were investigated [77]. While blood TG levels were higher in the early stages of hyperlipidemia, a decrease in TG levels was observed at a later stage. This decrease was suggested to be either a consequence of liver adaptation to a high-lipid diet or natural regulation of hepatic lipid metabolism. In addition, as shown in Table 3, differences in metabolite levels in the rat liver were observed at different stages of the experiment. Moreover, most differential metabolites were elevated in the hyperlipidemia group, which suggests that high lipid levels in the liver may have led to the accumulation of metabolites. 

**Table 3:** Summary of metabolomic studies reporting alterations in differential metabolites associated with hyperlipidemia.

Sample Type	Analytical Technique	Statistical Details	Sample Source	Potential Biomarkers		References
Human (2016)	UPLC-MS	Orthogonal partial least squares discriminant analysis (OPLS-DA), *t*-test (*p* value of 0.05 or less)	Serum	**↑**	1-(sn-glycero-3-phospho)-1d-myo-inositol, Gamma-Glutamyl-beta-cyanoalanine, Uric acid, Beta-D-Galactose, Acetyl-N-formyl-5-methoxykynurenamine, P-Cresol, Azelaic acid, N-[(3a,5b,7a)-3-Hydroxy-24-oxo-7-(sulfooxy)cholan-24-yl]-glycine, 2-Phenylethanol glucuronide, Murocholic acid, Sphingosine 1-phosphate, LysoPC(18:3(6Z,9Z,12Z)), LysoPC(20:5(5Z,8Z,11Z,14Z,17Z)), LysoPC(18:3(9Z,12Z,15Z)), LysoPC(16:1(9Z)), LysoPC(22:6(4Z,7Z,10Z,13Z,16Z,19Z)), 14,15-Epoxy-5,8,11-eicosatrienoic acid, LysoPC(20:3(5Z,8Z,11Z)), LysoPC(16:0), LysoPC(22:5(7Z,10Z,13Z,16Z,19Z)), LysoPC(22:4(7Z,10Z,13Z,16Z)), LysoPC(15:0), LysoPC(18:0), LysoPC(P-18:0), Linoleic acid, PC(18:0/20:5(5Z,8Z,11Z,14Z,17Z)), Oleic acid, SM(d18:0/16:1(9Z), PC(18:0/18:4(6Z,9Z,12Z,15Z)), Chenodeoxycholic acid, and PE(14:1(9Z)/14:1(9Z)).	[21]
				**↓**	4-Hydroxybenzaldehyde, Testosterone sulfate, LysoPC(14:0), LysoPC(18:1(11Z)), LysoPC(P-16:0), and Maslinic acid.	
Human (2023)	^1^H-NMR	Partial least squares discriminant analysis (PLS-DA), Mann–Whitney U test (FDR-adjusted *p* value < 0.05), variable importance in projection (VIP) score ≥ 1	Plasma	**↑**	Tyrosine, glutamic acid, ornithine, lysine, alanine, creatinine, oxoglutaric acid, and creatine.	[66]
Human (2019)	UPLC-Q-TOF/MS	PLS-DA, VIP > 1, *t*-test (*p* value < 0.05)	Urine	**↑**	Prolylhydroxyproline, N-acetyltryptophan, L-Isoleucine, L-Homocystine, 5-Oxoproline, N-acetylglutamic acid, Betaine, Hydroxyphenylacetylglycine, Phenylacetic acid, Glutaric acid, Homovanillic acid sulfate, Dihyroxy-1H-indole glucuronide I, Porphobilinogen, Cortexolone, and Deoxyguanosine.	[64]
				**↓**	L-Proline, N-phenylacetylphenylalanine, L-Glutamine, Glycocholic acid, 2-Phenylglycine, Caproic acid, and Sebacic acid.	
Rat (2014)	UPLC-Q-TOF/MS	OPLS-DA, VIP > 1.5, independent sample *t*-test (*p* value < 0.05)	Serum	**↑**	Succinic acid, Cholic acid, C16 Sphinganine, and Sphinganine.	[68]
				**↓**	Stearidonic acid, Linoleic acid, 8,11-Eicosadiynoic acid, Eicosapentaenoic acid, and DHA.	
			Urine	↑	DHA, 3-methyluridine, uridine, L-isoleucine, and phenyllactic acid.	
				↓	Hippuric acid, taurine, L-cysteine, norepinephrine, and L-carnitine.	
Mice (2020)	^1^H-NMR	Principal component analysis (PCA), OPLS, VIP > 1, *t*-test (*p* value < 0.05)	Plasma	Eleven metabolites (glycerol, glucose, leucine, arginine, betaine, lysine, glutamine, glutamate, valine, alanine, and choline) had regular changes within nine points during the study.		[70]
Rat (2020)	LC-MS	One-way analysis of variance (ANOVA) followed by Turkey–Kramer multiple comparison test	Serum	↓	Alanine, arginine, lysine, methionine, serine, tyrosine, and valine.	[63]
Rat (2016)	GC-MS, LC-MS, and Capillary electrophoresis-mass spectrometry (CE-MS)	PCA, PLS-DA, OPLS-DA, Welch’s *t*-test (*p* value < 0.05), Benjamin Hochberg FDR correction	Plasma	**↑**	PE (O-36:4), PC (18:1/16:0), PC (35:2), LPE (16:0), LPE(18:0), LPE (18:1), LPE (18:2), LPC (15:0) sn-1, Glycocholic acid, Cholic acid, Deoxyvitamin D3, Dihydroxy-oxo-vitamin D3, Trinorvitamin D3 carboxylic acid, Oleoylcarnitine, Linoleoyl carnitine, Propanoylcarnitine, Cytosine, Cholesterol, Lysine, Glycine, Acetoacetate, Citric acid, Pyranoses, l-Serine, Glyceric acid, Acetyl-l-carnitine, Propionyl-l-carnitine, NG,NG-dimethyl-l-arginine, Kynurenine, Ornithine, Betaine, Isoputreanine, Nx-Acetylspermidine, Asparagine, 5-Hydroxylysine, Histidine, N-methyl-l-histidine, and Cytidine.	[67]
				**↓**	Arachidonic Acid, PE (P-19:1), PE-NMe2 (16:0/20:4), PE (P-38:6), PC (15:0), PC (34:4), PC (20:4/16:0), PC (36:4), PC (37:4), PC (20:4/18:0), PC (18:2/20:4), PC (40:5), PC (40:7), PC (40:8), PC (42:10), LPC (16:0), LPC (17:0), LPC (18:0) sn-1, LPC (P-18:1) sn-1, LPC (19:0) sn-2, LPC (20:4) sn-2, LPC (22:5) sn-1, LPC (22:6) sn-1, SM (32:1), SM (33:1), SM (34:2), SM (34:1), PI-Cer (40:1), Carnitine, Linoleoyl taurine, Leucyl-proline, Bilirubin, 2-Ketoisocaproic acid, D-Galactopyranoside, Glycerol, Arginine, and Cysteine–homocysteine disulfide.	
Rat (2014)	UPLC-Q-TOF/HDMS	OPLS-DA, ANOVA followed by *t*-test for multiple comparisons (*p* < 0.05)	Urine	**↑**	Octadecanamide, Oleamide, Tryptophan, Ursodeoxycholic acid, Creatinine, Ascorbalamic acid, 3-Methyluridine, Indole-3-carboxylic Acid, and Tryptophyl-tyrosine.	[73]
				**↓**	Citric acid, Adenosine 2′,3′-cyclic phosphate, 3-O-Methyldopa, Proline, 1-Methyladenosine, Phenylalanine, and 5-Methylcytosine.	
Rat (2023)	GC-MS and LC/MS/MS	Welch’s two-sample *t*-tests (*p* value ≤ 0.05)	Aorta	**↑**	30 differential metabolites.	[76]
				**↓**	40 differential metabolites.	
			Heart	**↑**	122 differential metabolites.	
				**↓**	59 differential metabolites.	
			Liver	**↑**	67 differential metabolites.	
				**↓**	78 differential metabolites.	
			Plasma	**↑**	97 differential metabolites.	
				**↓**	75 differential metabolites.	
Rat (2023)	MSI	PLS-DA, volcano plot, VIP > 1, fold change > 1.5 or <0.75, *p* value < 0.05	Liver	PA (20:3-OH/i-21:0), PA (20:4-OH/22:6), PG (20:5-OH/i-16:0), PG (22:6-2OH/i-13:0), PG(O-18:0/20:4), PGP (18:3-OH/i-12:0), PGP(PGJ2/i-15:0), SM(d18:0/18:1-2OH), and TG (14:0/14:0/16:0) showed differences through the entire experimental period.		[77]

## 4. Metabolomics as a Tool for the Investigation of the Activity of Therapeutic Agents

### 4.1. Conventional Anti-Hyperlipidemic Drugs

Several lipid-lowering agents function through different mechanisms to regulate lipid levels. Statins are considered first-line agents for hyperlipidemia and are among the most frequently used lipid-lowering drugs [24,78]. Statins are competitive inhibitors of the rate-limiting enzyme of cholesterol synthesis, HMG-CoA reductase [79]. HMG-CoA reductase converts HMG-CoA to mevalonate, and, by blocking this enzyme, statins can reduce the hepatic synthesis of cholesterol, subsequently increasing the expression of LDL-C receptors and promoting the uptake of circulating LDL-C into hepatocytes, all of which contribute to lipid-lowering effects [79,80]. In recent years, metabolomic studies have focused on the underlying metabolic alterations induced by statin administration to provide profound insights into their mechanisms. 

The mechanism underlying the effects of simvastatin on the gut microbiota and metabolic profiles has recently been investigated. Using ultrahigh-performance liquid chromatography coupled with hybrid triple quadrupole time-of-flight mass spectrometry (UHPLC-Q-TOF MS/MS)-based metabolomics, the serum metabolites after the simvastatin treatment in rats with diet-induced hyperlipidemia were analyzed [81]. Six endogenous metabolites (m-coumaric acid, DL-phenylalanine, 3-(2-hydroxyphenyl) propionic acid, L-tyrosine, linoleic acid, and 9-(R)-hydroxyoctadecadienoic acid) were altered following the simvastatin administration. The simvastatin treatment affected the metabolism of amino acids and unsaturated fatty acids and the functions of gut microbial metabolism. Moreover, the affected metabolic pathways showed a strong correlation with the altered gut microbiota investigated in this study. In 2022, another study focused on the metabolic signature of simvastatin by performing an LC-MS/MS metabolomic analysis of 1332 participants treated with simvastatin and 6200 participants not treated with statins [82]. A total of 321 metabolites were significantly altered by the simvastatin treatment. In addition, among these metabolites, 313 were considered novel and previously unpublished in association with simvastatin treatment. The findings of this study illustrate that simvastatin treatment has a more complex metabolic signature, affecting various metabolic pathways, including lipids, amino acids, peptides, nucleotides, carbohydrates, cofactors, vitamins, and xenobiotics. Another study reported the effects of simvastatin on hyperlipidemia, specifically on hepatic lipid metabolism and the composition of the intestinal microbiota in rats [83]. The promising outcomes of simvastatin treatment include decreased blood and hepatic cholesterol levels, enhanced lipid metabolism, and beneficial modifications to the composition of the gut microbiota in hyperlipidemic rats. In hyperlipidemia, certain metabolites display altered levels. Among the metabolites that increase in response to disease are linoleic acid, arachidonic acid, and DHA. The metabolic abnormalities linked to hyperlipidemia are reflected in these variations in metabolite concentrations, which also offer insights into the causes of the disease and possible targets for treatment [83]. 

A multi-omics investigation involving fecal- and plasma-based metabolomic analyses of atorvastatin administration in HFD rats was conducted [84]. The ability of atorvastatin to improve high plasma lipid levels was reported to occur through the regulation of propanoate metabolism and glycine, serine, and threonine metabolism in feces and the plasma metabolome. Additionally, atorvastatin affected pantothenate and CoA biosynthesis and valine, leucine, and isoleucine biosynthesis in the plasma metabolome. These alterations were attributed to the effects of atorvastatin on intestinal microbes, mainly by increasing the abundance of Bacteroides. 

Based on the findings of the metabolomic-based studies of statins, the lipid-lowering activity of statins may be attributed to their role in the regulation of the gut microbiota and several metabolic pathways with amino acid metabolism and fatty acid metabolism, which are commonly influenced by statin administration.

### 4.2. Traditional Herbal Products in Hyperlipidemia Treatment

Although several lipid-lowering drugs with different mechanisms of action are available on the market, problems associated with their side effects and lack of efficacy have limited their use [85]. Several studies have focused on testing natural agents as alternative treatments for the prevention and treatment of hyperlipidemia [86]. Although several herbal agents have been reported to exhibit lipid-lowering activity, the exact mechanism of action of these agents remains unclear. Metabolomics offers a tool for evaluating the efficacy of natural products and elucidating their mechanisms of action [87]. 

Yellowhorn tea (YT), a tea obtained from the leaves of the traditional Chinese medicinal (TCM) herb Xanthoceras sorbifolium Bunge, is known for its lipid-lowering effects [88]. Liver-based untargeted metabolomics was performed using LC-MS/MS to determine the underlying metabolic changes in YT in HFD-induced hyperlipidemic mice [32]. A total of 21 metabolites were selected as potential biomarkers, and the administration of YT reversed the abnormal levels of 12 of these metabolites back to normal, demonstrating the role of YT in the amelioration of HFD-induced hyperlipidemia, oxidative stress, and inflammation. The YT extract was found to alleviate hyperlipidemia, possibly through the regulation of three metabolic pathways, including glycerophospholipid metabolism, vitamin B6 metabolism, and nicotinate and nicotinamide metabolism. 

Hawthorn extract, a TCM product, has been reported to exhibit lipid-lowering activity [89,90]. Two studies investigated the metabolic changes following hawthorn administration in HFD-hyperlipidemic rats using a metabolomics approach. Using a GC-MS analysis, the administration of hawthorn ethanol extract was observed to partially modulate the disturbance in metabolic pathways induced by the development of hyperlipidemia [91]. Of the 15 identified potential biomarkers associated with hyperlipidemia, the hawthorn ethanol extract elevated the levels of threonine, aspartic acid, glutamine, mannose, inositol, and oleic acid in the adipose tissue of HFD rats. Recently, another study assessed the efficacy of four different polar parts of the hawthorn using LC-MS plasma-based metabolomics [33]. A total of 22 potential biomarkers of hyperlipidemia were selected, which mainly included amnio acids, lysophosphatidylcholines, unsaturated fatty acids, and cholic acid. The most effective fraction ameliorated a disordered metabolism by regulating lipid metabolism, energy metabolism, oxidative stress, and amino acid metabolism and restored the biomarker levels to normal levels.

An untargeted urine metabolomic analysis of the effect of Citri Reticulatae Chachiensis Pericarpium (CRCP) in HFD-induced hyperlipidemic rats using an UPLC-Q-TOF/MS analysis demonstrated that the lipid-lowering activity of CRCP occurs mainly by significantly restoring the levels of 5-L-glutamyl-taurine, 5-aminopentanoic acid, cis-4-octenedioic acid, and 2-octenedioic acid back to normal, compared to the elevated levels observed in HFD rats [92]. These findings suggest that CRCP exerts its hypolipidemic effect through the regulation of taurine and hypotaurine metabolism, fatty acid biosynthesis, and arginine and proline metabolism [92].

Ilicis Rotundae Cortex (IRC), the dried bark of Ilex rotunda Thunb, is used in TCM for the treatment of CVDs. The lipid-lowering activity of the bioactive components in IRC has recently been reported [93,94]. A study conducted using ultraperformance liquid chromatography–quadrupole time-of-flight tandem mass spectrometry (UPLC-Q-TOF-MS/MS)-based metabolic profiling in HFD rats explored the anti-hyperlipidemic activity of the IRC extract [95]. The altered levels of 62 metabolites were observed, including 23 differential metabolites in the plasma, 26 in the urine, and 15 in the cecum content. These metabolites included amino acids, glycerophospholipids, sphingolipids, fatty acids, and bile acids. The results showed that the IRC lipid-lowering effect occurred mainly through the partial reversal of the disturbance of bile acid metabolism, linoleic acid metabolism, arachidonic acid metabolism, taurine and hypotaurine metabolism, glyoxylate and dicarboxylate metabolism, glycerophospholipid metabolism, the synthesis and degradation of ketone bodies, sphingolipid metabolism, and riboflavin metabolism. 

## 5. Limitations and Future Applications

Metabolomics is widely used in clinical and basic studies owing to its applications in disease diagnostics, therapeutic efficacy evaluation, and pathological processes. However, there are still various challenges and limitations to current metabolomic studies in hyperlipidemia. This current review includes a diverse range of experiments aimed at identifying a potential biomarker using mainly untargeted metabolomics strategies to better understand the disease. Even though the number of studies exploring biomarkers in hyperlipidemia models is limited and considered one of the biggest challenges in this topic, the existing research provides valuable insights into potential diagnostic and therapeutic targets. Moreover, there is variation in the methodologies, such as the protocols used for the sample preparation, in the experimental design, and in the techniques used across metabolomic studies. In addition, there are challenges from the generalizability of the findings, because most clinical metabolomics studies on hyperlipidemic subjects have been conducted on certain populations and relatively small sample sizes. While existing studies provide valuable insights into hyperlipidemia and the activity of lipid-lowering agents, expanding the research to more diverse populations using larger and more representative samples could enhance the generalizability of the findings and further facilitate the validation of novel biomarkers.

Precision medicine is an innovative approach that tailors therapeutic strategies based on an individual’s genetic characteristics and environmental and lifestyle factors [96]. Since changes in the genomic, transcriptomic, and proteomic levels are reflected as metabolic alterations, metabolomics has emerged as a useful approach that facilitates the application of precision medicine in various diseases [97,98]. As part of future work, hyperlipidemia research could benefit from a precision medicine approach, employing the findings of the metabolomics studies conducted on hyperlipidemia models.

## 6. Conclusions

Hyperlipidemia is a major public health concern. It is a significant risk factor for cardiovascular diseases and other health complications. In this review, we examined the key metabolites related to hyperlipidemia. These metabolites can be used as indicators in metabolomic investigations of hyperlipidemia and may be a focus of future studies. By examining metabolomics-based studies on hyperlipidemia, we identified the key pathways that are consistently affected under hyperlipidemic conditions. Lipid metabolism, energy metabolism, the TCA cycle, and amino acid metabolism are the disrupted metabolic pathways impacted by hyperlipidemia. Furthermore, metabolomics has emerged as a useful approach for investigating the metabolic mechanisms underlying the lipid-lowering activity of therapeutic agents. This review provides insights into the treatment of hyperlipidemia. To further enhance our understanding, additional research is needed to determine whether the identified differential metabolites can be utilized as diagnostic tools and to elucidate the molecular mechanisms underlying the changes in metabolite levels during metabolic disorders.

## Figures and Tables

**Table 2:** Strategies applied in the search for metabolomic-based studies on hyperlipidemia.

Methodology	
Databases	Google Scholar, PubMed, and ScienceDirect
Terms used in the search process	“hyperlipidemia” AND (“metabolomics” OR “metabolic profiling”) AND (“tissue” OR “plasma” OR “serum” OR “urine”) AND (“biomarker” OR “potential biomarker”)
Included articles	Articles focusing on biomarker identification and metabolite level alteration in hyperlipidemia
Filters	Articles published from 2014 to 2024

## Data Availability

The data are contained within the article.

## References

[1] Karr S. (2017). Epidemiology and management of hyperlipidemia. Am. J. Manag. Care.

[2] Hill M.F., Bordoni B. (2022). Hyperlipidemia. StatPearls.

[3] Nouh F., Omar M., Younis M. (2018). Risk factors and management of hyperlipidemia. Asian J. Cardiol. Res..

[4] Rachitha P., Krishnaswamy K., Lazar R.A., Gupta V.K., Inbaraj B.S., Raghavendra V.B., Sharma M., Sridhar K. (2023). Attenuation of hyperlipidemia by medicinal formulations of Emblica officinalis synergized with nanotechnological approaches. Bioengineering.

[5] Bilen O., Pokharel Y., Ballantyne C.M. (2016). Genetic testing in hyperlipidemia. Endocrinol. Metab. Clin..

[6] Ezeh K.J., Ezeudemba O. (2021). Hyperlipidemia: A review of the novel methods for the management of lipids. Cureus.

[7] Kokkinos P., Katsagoni C.N., Sidossis L.S. (2023). Prevention and Management of Cardiovascular and Metabolic Disease: Diet, Physical Activity and Healthy Aging.

[8] Mumthaj P., Natarajan P., Janani A., Vijay J., Gokul V. (2021). A Global Review Article on Hyperlipidemia. Int. J. Pharm. Sci. Rev. Res..

[9] Amini M., Zayeri F., Salehi M. (2021). Trend analysis of cardiovascular disease mortality, incidence, and mortality-to-incidence ratio: Results from global burden of disease study 2017. BMC Public Health.

[10] Karam I., Ma N., Liu X., Li J., Yang Y. (2019). Short Review on Hyperlipidemia. J. Blood Transfus. Dis..

[11] Di Cesare M., Perel P., Taylor S., Kabudula C., Bixby H., Gaziano T.A., McGhie D.V., Mwangi J., Pervan B., Narula J. (2024). The Heart of the World. Glob. Heart.

[12] Şahin B., İlgün G. (2022). Risk factors of deaths related to cardiovascular diseases in World Health Organization (WHO) member countries. Health Soc. Care Community.

[13] Mensah G.A., Fuster V., Murray C.J.L., Roth G.A. (2023). Global burden of cardiovascular diseases and risks 1990–2022. J. Am. Coll. Cardiol..

[14] Frąk W., Wojtasińska A., Lisińska W., Młynarska E., Franczyk B., Rysz J. (2022). Pathophysiology of Cardiovascular Diseases: New Insights into Molecular Mechanisms of Atherosclerosis, Arterial Hypertension, and Coronary Artery Disease. Biomedicines.

[15] Wang T., Butany J. (2017). Pathogenesis of atherosclerosis. Diagn. Histopathol..

[16] Bui Q.T., Prempeh M., Wilensky R.L. (2009). Atherosclerotic plaque development. Int. J. Biochem. Cell Biol..

[17] Wouters K., Shiri-Sverdlov R., van Gorp P.J., van Bilsen M., Hofker M.H. (2005). Understanding hyperlipidemia and atherosclerosis: Lessons from genetically modified apoe and ldlr mice. Clin. Chem. Lab. Med. (CCLM).

[18] Rafieian-Kopaei M., Setorki M., Doudi M., Baradaran A., Nasri H. (2014). Atherosclerosis: Process, indicators, risk factors and new hopes. Int. J. Prev. Med..

[19] Liu Y., Zhang Q., Zhao G., Liu G., Liu Z. (2020). Deep learning-based method of diagnosing hyperlipidemia and providing diagnostic markers automatically. Diabetes Metab. Syndr. Obes..

[20] Lin C., Tian Q., Guo S., Xie D., Cai Y., Wang Z., Chu H., Qiu S., Tang S., Zhang A. (2024). Metabolomics for clinical biomarker discovery and therapeutic target identification. Molecules.

[21] Zhao Q., Zhang A., Zong W., An N., Zhang H., Luan Y., Cao H., Sun H., Wang X. (2016). Chemometrics strategy coupled with high resolution mass spectrometry for analyzing and interpreting comprehensive metabolomic characterization of hyperlipemia. RSC Adv..

[22] Dong W., Zhang F., Lian D., Chen X., Zhou H., Gong T., Wang C. (2022). Efficacy and safety of tai chi for hyperlipidaemia: A protocol for systematic review and meta-analysis. BMJ Open.

[23] Su L., Mittal R., Ramgobin D., Jain R., Jain R. (2021). Current management guidelines on hyperlipidemia: The silent killer. J. Lipids.

[24] Stewart J., McCallin T., Martinez J., Chacko S., Yusuf S. (2020). Hyperlipidemia. Pediatr. Rev..

[25] Poznyak A.V., Zhang D., Orekhova V., Grechko A.V., Wetzker R., Orekhov A.N. (2020). A brief overview of currently used atherosclerosis treatment approaches targeting lipid metabolism alterations. Am. J. Cardiovasc. Dis..

[26] Alwahsh M., Knitsch R., Marchan R., Lambert J., Hoerner C., Zhang X., Schalke B., Lee D.-H., Bulut E., Graeter T. (2022). Metabolic profiling of thymic epithelial tumors hints to a strong Warburg Effect, glutaminolysis and precarious redox homeostasis as potential therapeutic targets. Cancers.

[27] Srivastava S. (2019). Emerging insights into the metabolic alterations in aging using metabolomics. Metabolites.

[28] Nalbantoglu S. (2019). Metabolomics: Basic principles and strategies. Mol. Med..

[29] Chen H., Miao H., Feng Y.-L., Zhao Y.-Y., Lin R.-C. (2014). Metabolomics in dyslipidemia. Adv. Clin. Chem..

[30] Zhang X., Zhu X., Wang C., Zhang H., Cai Z. (2016). Non-targeted and targeted metabolomics approaches to diagnosing lung cancer and predicting patient prognosis. Oncotarget.

[31] Bingol K. (2018). Recent advances in targeted and untargeted metabolomics by NMR and MS/NMR methods. High-Throughput.

[32] Ta N., Lisha A., Erdunduleng E., Qi R., Mu X., Feng L., Ba G., Li Y., Zhang J., Bai L. (2023). Metabolomics analysis reveals amelioration effects of yellow horn tea extract on hyperlipidemia, inflammation, and oxidative stress in high-fat diet-fed mice. Front. Nutr..

[33] Zeng L., Luo L., Xue Q., He Q., Chen X., Meng J., Wang S., Liang S. (2021). LC–MS based plasma metabolomics study of the intervention effect of different polar parts of Hawthorn on hyperlipidemia rats. J. Sep. Sci..

[34] Barnes S., Benton H.P., Casazza K., Cooper S.J., Cui X., Du X., Engler J., Kabarowski J.H., Li S., Pathmasiri W. (2016). Training in metabolomics research. II. Processing and statistical analysis of metabolomics data, metabolite identification, pathway analysis, applications of metabolomics and its future. J. Mass Spectrom..

[35] Monteiro M., Carvalho M., Bastos M., Guedes de Pinho P. (2013). Metabolomics analysis for biomarker discovery: Advances and challenges. Curr. Med. Chem..

[36] Johnson C.H., Ivanisevic J., Siuzdak G. (2016). Metabolomics: Beyond biomarkers and towards mechanisms. Nat. Rev. Mol. Cell Biol..

[37] Fraga-Corral M., Carpena M., Garcia-Oliveira P., Pereira A., Prieto M., Simal-Gandara J. (2022). Analytical metabolomics and applications in health, environmental and food science. Crit. Rev. Anal. Chem..

[38] Alhusban A.A., Albustanji S., Hamadneh L.A., Shallan A.I. (2021). High performance liquid chromatography–tandem mass spectrometry method for correlating the metabolic changes of lactate, pyruvate and L-glutamine with induced tamoxifen resistant MCF-7 cell line potential molecular changes. Molecules.

[39] Fonseca T.A., Oliveira M.C., Araújo R., Bento L., Von Rekowski C., Justino G.C., Calado C.R. Comparison of Analytical Methods Of Serum Untargeted Metabolomics. Proceedings of the 2023 IEEE 7th Portuguese Meeting on Bioengineering (ENBENG).

[40] Sivamani Y., Murthy K.N., Sajal H., Elayaperumal S., Sarma H., Joshi S., Lahiri D., Ray R.R., Davoodbasha M. (2023). Chapter 13—Isotope labeling LC-MS for metabolomics of biofilm study and tracer-based biofilm metabolomics analysis. Microbial Biofilms.

[41] Chen X., Shu W., Zhao L., Wan J. (2023). Advanced mass spectrometric and spectroscopic methods coupled with machine learning for in vitro diagnosis. View.

[42] Miguez A.M., Zhang Y., Styczynski M. (2022). Metabolomics Analysis of Cell-Free Expression Systems Using Gas Chromatography-Mass Spectrometry. Cell-Free Gene Expression: Methods and Protocols.

[43] Wang Y., Liu S., Hu Y., Li P., Wan J.-B. (2015). Current state of the art of mass spectrometry-based metabolomics studies—A review focusing on wide coverage, high throughput and easy identification. RSC Adv..

[44] Segers K., Declerck S., Mangelings D., Heyden Y.V., Eeckhaut A.V. (2019). Analytical techniques for metabolomic studies: A review. Bioanalysis.

[45] Silva C., Perestrelo R., Silva P., Tomás H., Câmara J.S. (2019). Breast cancer metabolomics: From analytical platforms to multivariate data analysis. A review. Metabolites.

[46] Emwas A.-H., Roy R., McKay R.T., Tenori L., Saccenti E., Gowda G.N., Raftery D., Alahmari F., Jaremko L., Jaremko M. (2019). NMR spectroscopy for metabolomics research. Metabolites.

[47] Aggarwal S., Banerjee N., Parihari S., Roy J., Bojak K., Shah R. (2022). Metabolomics: Role in pathobiology and therapeutics of COVID-19. Multi-Pronged Omics Technologies to Understand COVID-19.

[48] Ozcelikay G., Kaya S., Ozkan E., Cetinkaya A., Nemutlu E., Kır S., Ozkan S. (2022). Sensor-based MIP technologies for targeted metabolomics analysis. TrAC Trends Anal. Chem..

[49] Paul A., de Boves Harrington P. (2021). Chemometric applications in metabolomic studies using chromatography-mass spectrometry. TrAC Trends Anal. Chem..

[50] Gonzalez-Dominguez A., Duran-Guerrero E., Fernandez-Recamales A., Lechuga-Sancho A.M., Sayago A., Schwarz M., Segundo C., Gonzalez-Dominguez R. (2017). An overview on the importance of combining complementary analytical platforms in metabolomic research. Curr. Top. Med. Chem..

[51] Jeppesen M.J., Powers R. (2023). Multiplatform untargeted metabolomics. Magn. Reson. Chem..

[52] Chen Y., Li E.-M., Xu L.-Y. (2022). Guide to metabolomics analysis: A bioinformatics workflow. Metabolites.

[53] Sussulini A. (2017). Metabolomics: From Fundamentals to Clinical Applications.

[54] Aretz I., Meierhofer D. (2016). Advantages and pitfalls of mass spectrometry based metabolome profiling in systems biology. Int. J. Mol. Sci..

[55] Califf R.M. (2018). Biomarker definitions and their applications. Exp. Biol. Med..

[56] Zhang A., Sun H., Yan G., Wang P., Wang X. (2015). Metabolomics for biomarker discovery: Moving to the clinic. Biomed. Res. Int..

[57] Chen H., Yuan B., Miao H., Tan Y., Bai X., Zhao Y.-Y., Wang Y. (2015). Urine metabolomics reveals new insights into hyperlipidemia and the therapeutic effect of rhubarb. Anal. Methods.

[58] Rai S., Bhatnagar S. (2017). Novel lipidomic biomarkers in hyperlipidemia and cardiovascular diseases: An integrative biology analysis. Omics J. Integr. Biol..

[59] German C.A., Shapiro M.D. (2020). Assessing atherosclerotic cardiovascular disease risk with advanced lipid testing: State of the science. Eur. Cardiol. Rev..

[60] Xu Q.-y., Liu Y.-h., Zhang Q., Ma B., Yang Z.-d., Liu L., Yao D., Cui G.-b., Sun J.-j., Wu Z.-m. (2014). Metabolomic analysis of simvastatin and fenofibrate intervention in high-lipid diet-induced hyperlipidemia rats. Acta Pharmacol. Sin..

[61] O’Neill S., Bohl M., Gregersen S., Hermansen K., O’Driscoll L. (2016). Blood-based biomarkers for metabolic syndrome. Trends Endocrinol. Metab..

[62] Kalyani A., Jha R.M., Sharma S. (2019). Use of circulating nucleic acids, metabolites, and proteins as clinical biomarkers for earlier prognosis and diagnosis of disease. Prognostic Epigenetics.

[63] Wang X.-F., Zhang Y.-X., Ma H.-Y. (2020). Targeted profiling of amino acid metabolome in serum by a liquid chromatography-mass spectrometry method: Application to identify potential markers for diet-induced hyperlipidemia. Anal. Methods.

[64] Yang L., Li Z., Song Y., Liu Y., Zhao H., Liu Y., Zhang T., Yuan Y., Cai X., Wang S. (2019). Study on urine metabolic profiling and pathogenesis of hyperlipidemia. Clin. Chim. Acta.

[65] Li Q., Gu W., Ma X., Liu Y., Jiang L., Feng R., Liu L. (2016). Amino acid and biogenic amine profile deviations in an oral glucose tolerance test: A comparison between healthy and hyperlipidaemia individuals based on targeted metabolomics. Nutrients.

[66] Gu P.-S., Su K.-W., Yeh K.-W., Huang J.-L., Lo F.-S., Chiu C.-Y. (2023). Metabolomics Analysis Reveals Molecular Signatures of Metabolic Complexity in Children with Hypercholesterolemia. Nutrients.

[67] González-Peña D., Dudzik D., Colina-Coca C., de Ancos B., García A., Barbas C., Sánchez-Moreno C. (2016). Multiplatform metabolomic fingerprinting as a tool for understanding hypercholesterolemia in Wistar rats. Eur. J. Nutr..

[68] Wu Q., Zhang H., Dong X., Chen X.-F., Zhu Z.-Y., Hong Z.-Y., Chai Y.-F. (2014). UPLC-Q-TOF/MS based metabolomic profiling of serum and urine of hyperlipidemic rats induced by high fat diet. J. Pharm. Anal..

[69] Liu X., Yu J., Zhao J., Guo J., Zhang M., Liu L. (2020). Glucose challenge metabolomics implicates the change of organic acid profiles in hyperlipidemic subjects. Biomed. Chromatogr..

[70] Li Y., Zhao X.-J. (2020). NMR-based plasma metabonomics in hyperlipidemia mice. Anal. Methods.

[71] Chen Y.-L., Xiao C.-H., Hu Z.-X., Liu X.-S., Liu Z., Zhang W.-N., Zhao X.-J. (2017). Dynamic lipid profile of hyperlipidemia mice. J. Chromatogr. B.

[72] Jedinak A., Loughlin K.R., Moses M.A. (2018). Approaches to the discovery of non-invasive urinary biomarkers of prostate cancer. Oncotarget.

[73] Miao H., Chen H., Zhang X., Yin L., Chen D.-Q., Cheng X.-L., Bai X., Wei F. (2014). Urinary metabolomics on the biochemical profiles in diet-induced hyperlipidemia rat using ultraperformance liquid chromatography coupled with quadrupole time-of-flight SYNAPT high-definition mass spectrometry. J. Anal. Methods Chem..

[74] Jin W., Li C., Yang S., Song S., Hou W., Song Y., Du Q. (2023). Hypolipidemic effect and molecular mechanism of ginsenosides: A review based on oxidative stress. Front. Pharmacol..

[75] Saoi M., Britz-McKibbin P. (2021). New advances in tissue metabolomics: A review. Metabolites.

[76] Xu K., Saaoud F., Shao Y., Lu Y., Wu S., Zhao H., Chen K., Vazquez-Padron R., Jiang X., Wang H. (2023). Early hyperlipidemia triggers metabolomic reprogramming with increased SAH, increased acetyl-CoA-cholesterol synthesis, and decreased glycolysis. Redox Biol..

[77] Mao H., Wang W., Xiang X., Li Y., Zhao J., Huang Y., Di S., Zhuo Q., Nie H. (2023). Analysis of metabolite distribution in rat liver of high-fat model by mass spectrometry imaging. Metabolites.

[78] Feingold K.R. (2016). Cholesterol Lowering Drugs.

[79] Sizar O., Khare S., Jamil R.T., Talati R. (2017). Statin Medications.

[80] Fiorentino R., Chiarelli F. (2023). Statins in Children, an Update. Int. J. Mol. Sci..

[81] Zhang S., Yuan L., Li H., Han L., Jing W., Wu X., Ullah S., Liu R., Wu Y., Xu J. (2021). The novel interplay between commensal gut bacteria and metabolites in diet-induced hyperlipidemic rats treated with simvastatin. J. Proteome Res..

[82] Fernandes Silva L., Ravi R., Vangipurapu J., Laakso M. (2022). Metabolite signature of simvastatin treatment involves multiple metabolic pathways. Metabolites.

[83] Zhang Q., Fan X., Ye R., Hu Y., Zheng T., Shi R., Cheng W., Lv X., Chen L., Liang P. (2020). The effect of simvastatin on gut microbiota and lipid metabolism in hyperlipidemic rats induced by a high-fat diet. Front. Pharmacol..

[84] Li H., Wang S., Wang S., Yu H., Yu W., Ma X., He X. (2022). Atorvastatin inhibits high-fat diet-induced lipid metabolism disorders in rats by inhibiting Bacteroides reduction and improving metabolism. Drug Des. Dev. Ther..

[85] Abu Farha R., Bustanji Y., Al-Hiari Y., Al-Qirim T., Abu Shiekha G., Albashiti R. (2016). Lipid lowering activity of novel N-(benzoylphenyl) pyridine-3-carboxamide derivatives in Triton WR-1339-induced hyperlipidemic rats. J. Enzym. Inhib. Med. Chem..

[86] El-Tantawy W.H., Temraz A. (2019). Natural products for controlling hyperlipidemia. Arch. Physiol. Biochem..

[87] Wu G., Zhang W., Li H. (2019). Application of metabolomics for unveiling the therapeutic role of traditional Chinese medicine in metabolic diseases. J. Ethnopharmacol..

[88] Zang E., Qiu B., Chen N., Li C., Liu Q., Zhang M., Liu Y., Li M. (2021). *Xanthoceras sorbifolium* Bunge: A review on botany, phytochemistry, pharmacology, and applications. Front. Pharmacol..

[89] Shao F., Gu L., Chen H., Liu R., Huang H., Chen L., Yang M. (2017). Evaluation of hypolipidemic and antioxidant effects in phenolrich fraction of *Crataegus pinnatifida* fruit in hyperlipidemia rats and identification of chemical composition by ultra-performance liquid chromatography coupled with quadropole time-of-flight mass spectrometry. Pharmacogn. Mag..

[90] Dehghani S., Mehri S., Hosseinzadeh H. (2019). The effects of *Crataegus pinnatifida* (Chinese hawthorn) on metabolic syndrome: A review. Iran. J. Basic Med. Sci..

[91] Hu C., Zhang Y., Liu G., Liu Y., Wang J., Sun B. (2019). Untargeted metabolite profiling of adipose tissue in hyperlipidemia rats exposed to hawthorn ethanol extracts. J. Food Sci..

[92] Zeng W., Huang K.E., Luo Y., Li D.X., Chen W., Yu X.Q., Ke X.H. (2020). Nontargeted urine metabolomics analysis of the protective and therapeutic effects of *Citri reticulatae chachiensis pericarpium* on high-fat feed-induced hyperlipidemia in rats. Biomed. Chromatogr..

[93] Yang B., Li H., Ruan Q., Xuan S., Chen X., Cui H., Liu Z., Jin J., Zhao Z. (2019). Effects of gut microbiota and ingredient-ingredient interaction on the pharmacokinetic properties of rotundic acid and pedunculoside. Planta Medica.

[94] Liu C., Shen Y.-J., Tu Q.-B., Zhao Y.-R., Guo H., Wang J., Zhang L., Shi H.-W., Sun Y. (2018). Pedunculoside, a novel triterpene saponin extracted from Ilex rotunda, ameliorates high-fat diet induced hyperlipidemia in rats. Biomed. Pharmacother..

[95] Yang B., Xuan S., Ruan Q., Jiang S., Cui H., Zhu L., Luo X., Jin J., Zhao Z. (2020). UPLC/Q-TOF-MS/MS-based metabolomics revealed the lipid-lowering effect of Ilicis Rotundae Cortex on high-fat diet induced hyperlipidemia rats. J. Ethnopharmacol..

[96] Sethi Y., Patel N., Kaka N., Kaiwan O., Kar J., Moinuddin A., Goel A., Chopra H., Cavalu S. (2023). Precision medicine and the future of cardiovascular diseases: A clinically oriented comprehensive review. J. Clin. Med..

[97] Schmidt J.C., Dougherty B.V., Beger R.D., Jones D.P., Schmidt M.A., Mattes W.B. (2021). Metabolomics as a truly translational tool for precision medicine. Int. J. Toxicol..

[98] Gonzalez-Covarrubias V., Martínez-Martínez E., del Bosque-Plata L. (2022). The potential of metabolomics in biomedical applications. Metabolites.

